# Medical Education in a Global Pandemic: A Novel Case-Based Learning Approach to Teaching Plastic Surgery Topics to Preclerkship Students

**DOI:** 10.1177/22925503211054135

**Published:** 2021-11-25

**Authors:** Shaishav Datta, Chantal Valiquette, Kyle R Wanzel

**Affiliations:** 1 7938 Temerty Faculty of Medicine, University of Toronto, Toronto, ON, Canada; 2 Division of Plastic & Reconstructive Surgery, Department of Surgery, University of Toronto, Toronto, ON, Canada

There is currently limited structured curricular exposure to the diverse clinical practice of Plastic and Reconstructive Surgery (PRS) for Canadian medical students before their clerkship rotations. Most exposure during preclerkship years is limited to individually organized observerships. Even during clerkship, only a handful of students can participate in PRS core rotations before beginning electives. A 2015 survey conducted by our senior author found that >80% of all undergraduate medical students at our institution indicated that they had received no formal PRS teaching during their medical training.^1^ The same survey revealed that >44% of students felt that they would have considered a career in PRS if they had received exposure earlier in their training.^1^ Unfortunately, this lack of exposure has been further exacerbated by COVID-19, which greatly limited in-hospital opportunities for students. As such, it became important to develop an alternative method for students to gain exposure and find faculty and resident mentors in PRS. To meet this need, we created a PRS-focused, longitudinal, small-group, case-based learning (CBL) program, based on the outline by McKimm and Jones,^2^ that would allow preclerkship students the opportunity to learn about the various subspecialties of PRS. CBL was chosen as the teaching modality because it is an interactive educational paradigm that effectively links theory to practice using focused learning points.^3,4^ We compiled PRS topics and learning-objectives intended for clinical clerks designed by various Canadian institutions and used these to create a curriculum of nine CBL sessions (Table 1).^5-10^ These were delivered using live videoconferencing and utilized breakout sessions to engage small groups.

**Table 1.** List of topics and learning objectives used for PRS CBL program

Topics	Learning Objectives
Aesthetic surgery Breast reconstruction Burn surgery & care Complex wound closure Infection management Skin lesions Trauma (facial, upper extremity, & lower extremity)	Understand the role of a plastic surgeon in the healthcare team Describe the reconstructive ladder Understand steps of wound healing and relevant risk factors Demonstrate familiarity with clinical anatomy, including craniofacial, hand, and breast anatomy Recognize when to seek advice and consultation when managing a complex plastic surgery patient

A call for tutors at our institution's PRS department was sent in January 2021 and four residents, three fellows, and seven staff showed interest in creating teaching materials and facilitating sessions. The pilot program was held between February and June 2021 and was open to all first- and second-year medical students at our institution, with no expectation for prior PRS knowledge.

Initially, 7 students elected to participate in the program, but positive word-of-mouth reviews of the curriculum resulted in this number growing to 14 over one month. The sessions provided an opportunity for students to interact with PRS faculty and improve their basic knowledge of anatomy, clinical information gathering skills, and surgical decision-making. The small-group environment also provided space for students to receive career advice, mentorship, and establish ongoing relationships with tutors; as a result, two student-led research projects were initiated.

Moving forward, we plan to establish a year-long curriculum that will be examined through survey feedback. Despite established limitations of virtual teaching, such as “Zoom-fatigue,^11^” we found that the benefits of increased accessibility for students and tutors outweigh these limitations; and we plan to continue using this format in a postpandemic setting. We hope by sharing our endeavour, we can inspire similar interventions at other Canadian institutions. Through this, we believe that we can promote longitudinal engagement of medical students with PRS faculty, increase accessibility for students interested in PRS, and ultimately encourage students to consider a career in PRS.

**Figure 1.**
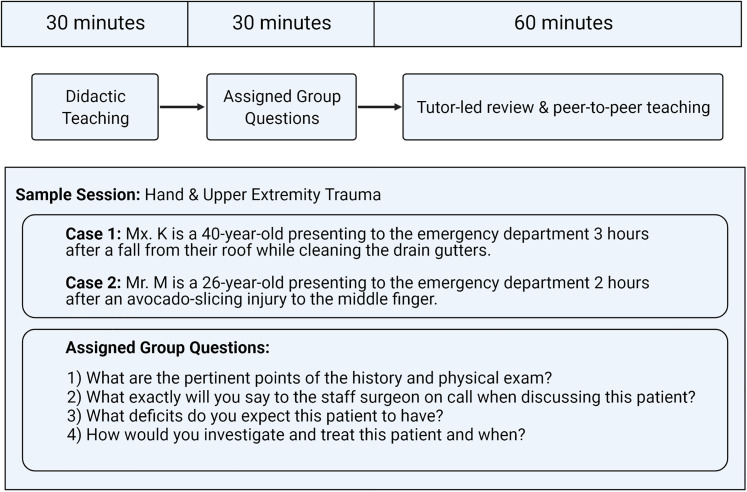
PRS CBL program structure with sample session plan.^3–9^ Created with BioRender.com
